# Risk Factors of *Streptococcus suis* Infection in Vietnam. A Case-Control Study

**DOI:** 10.1371/journal.pone.0017604

**Published:** 2011-03-08

**Authors:** Dang Trung Nghia Ho, Thi Phuong Tu Le, Marcel Wolbers, Quang Thai Cao, Van Minh Hoang Nguyen, Vu Thieu Nga Tran, Thi Phuong Thao Le, Hoan Phu Nguyen, Thi Hong Chau Tran, Xuan Sinh Dinh, Song Diep To, Thi Thanh Hang Hoang, Truong Hoang, James Campbell, Van Vinh Chau Nguyen, Tran Chinh Nguyen, Van Dung Nguyen, Thi Hoa Ngo, Brian G. Spratt, Tinh Hien Tran, Jeremy Farrar, Constance Schultsz

**Affiliations:** 1 Oxford University Clinical Research Unit, Wellcome Trust Major Overseas Programme, Hospital for Tropical Diseases, Ho Chi Minh City, Vietnam; 2 Pham Ngoc Thach University of Medicine, Ho Chi Minh City, Vietnam; 3 Hospital for Tropical Diseases, Ho Chi Minh City, Vietnam; 4 Sub-Department of Animal Health Ho Chi Minh City, Ho Chi Minh City, Vietnam; 5 Center for Poverty-related Communicable Diseases, Academic Medical Centre, Amsterdam Institute of Global Health and Development, University of Amsterdam, Amsterdam, The Netherlands; 6 Department of Infectious Disease Epidemiology, Imperial College London, London, United Kingdom; University of Iowa, United States of America

## Abstract

**Background:**

*Streptococcus suis* infection, an emerging zoonosis, is an increasing public health problem across South East Asia and the most common cause of acute bacterial meningitis in adults in Vietnam. Little is known of the risk factors underlying the disease.

**Methods and Findings:**

A case-control study with appropriate hospital and matched community controls for each patient was conducted between May 2006 and June 2009. Potential risk factors were assessed using a standardized questionnaire and investigation of throat and rectal *S. suis* carriage in cases, controls and their pigs, using real-time PCR and culture of swab samples. We recruited 101 cases of *S. suis* meningitis, 303 hospital controls and 300 community controls. By multivariate analysis, risk factors identified for *S. suis* infection as compared to either control group included eating “high risk” dishes, including such dishes as undercooked pig blood and pig intestine (OR_1_ = 2.22; 95%CI = [1.15–4.28] and OR_2_ = 4.44; 95%CI = [2.15–9.15]), occupations related to pigs (OR_1_ = 3.84; 95%CI = [1.32–11.11] and OR_2_ = 5.52; 95%CI = [1.49–20.39]), and exposures to pigs or pork in the presence of skin injuries (OR_1_ = 7.48; 95%CI = [1.97–28.44] and OR_2_ = 15.96; 95%CI = [2.97–85.72]). *S. suis* specific DNA was detected in rectal and throat swabs of 6 patients and was cultured from 2 rectal samples, but was not detected in such samples of 1522 healthy individuals or patients without *S. suis* infection.

**Conclusions:**

This case control study, the largest prospective epidemiological assessment of this disease, has identified the most important risk factors associated with *S. suis* bacterial meningitis to be eating ‘high risk’ dishes popular in parts of Asia, occupational exposure to pigs and pig products, and preparation of pork in the presence of skin lesions. These risk factors can be addressed in public health campaigns aimed at preventing *S. suis* infection.

## Introduction

The importance of zoonotic emerging infections is increasingly recognized, as illustrated by outbreaks of SARS coronavirus and the ongoing threat of human infections with avian influenza H5N1 virus. It is essential to understand transmission dynamics and risk factors for infection, in order to design effective strategies to contain and prevent the spread of zoonotic diseases [1]. *Streptococcus suis* infection is an emerging zoonotic infectious disease, which is increasingly reported in Asia. It has become an important threat for human health, illustrated by the explosive outbreak in Sichuan Province China associated with at least 215 cases and 39 deaths in 2005 [2]. In Vietnam, *S. suis* infection was first reported in 1996 and the number of human cases has increased annually. In Ho Chi Minh City and Hanoi, it causes approximately 40% of all adult acute bacterial meningitis cases. This is more than *Streptococcus pneumoniae* and *Neisseria meningitidis* combined [3], [4], [5].

*S. suis* is a Gram-positive, facultatively anaerobic coccus, which can be a commensal or pathogen for a wide range of mammalian species, particularly pigs. The natural habitat of *S. suis* in pigs is the upper respiratory-, the genital- and alimentary tracts. Based on differences in antigenic properties of the polysaccharide capsule, 33 serotypes have been distinguished to date, among which serotype 2 is most commonly associated with invasive disease in both pigs and humans. To date little is known of the risk factors underlying the disease or the portals of entry in humans. From small case series, the reported risk factor included occupational exposure, such as in slaughter house workers, butchers, and pig breeders, meat processing, and pig transport. It is hypothesized that patients may be infected through minor cuts or abrasions on their skin [2], [6]. However, whilst occupational exposure to pigs or pork was documented in 88% of the European patients described, it was reported in less than 50% of Asian cases [4], [7], suggesting the contribution of other behavioural or exposure related risk factors in Asian populations, such as culinary habits or close proximity of pigs within households. In addition, whilst it is known that pigs can carry *S. suis* asymptomatically, it is not known if there is asymptomatic carriage of *S. suis* in humans, which could potentially contribute to an increased risk of infection, and to the possibilitiy of person-to-person transmission. We conducted a prospective case-control study to identify the risk factors of *S. suis* infection in Viet Nam.

## Methods

### Ethical approval

The study was approved by the Scientific and Ethics Committee of the Hospital for Tropical diseases and the University of Oxford Tropical Research Ethics Committee (OXTREC 012-06).

### Study design and setting

This study was designed as a case-control study, including patients with invasive *S. suis* infection, an unmatched hospital control group and a community control group matched by residency and age, at a ratio of 1∶3. The study was conducted at the Hospital for Tropical Diseases (HTD), a tertiary referral hospital of infectious diseases in the south of Viet Nam, between May 2006 and June 2009. The recruitment of cases and hospital control groups took place at the dedicated Central Nervous System (CNS) infectious disease ward at HTD. Community controls were recruited according to the residency of the cases in the south of Viet Nam, mainly in Ho Chi Minh City and the provinces of the Mekong River Delta.

### Participants

**Figure 1:**
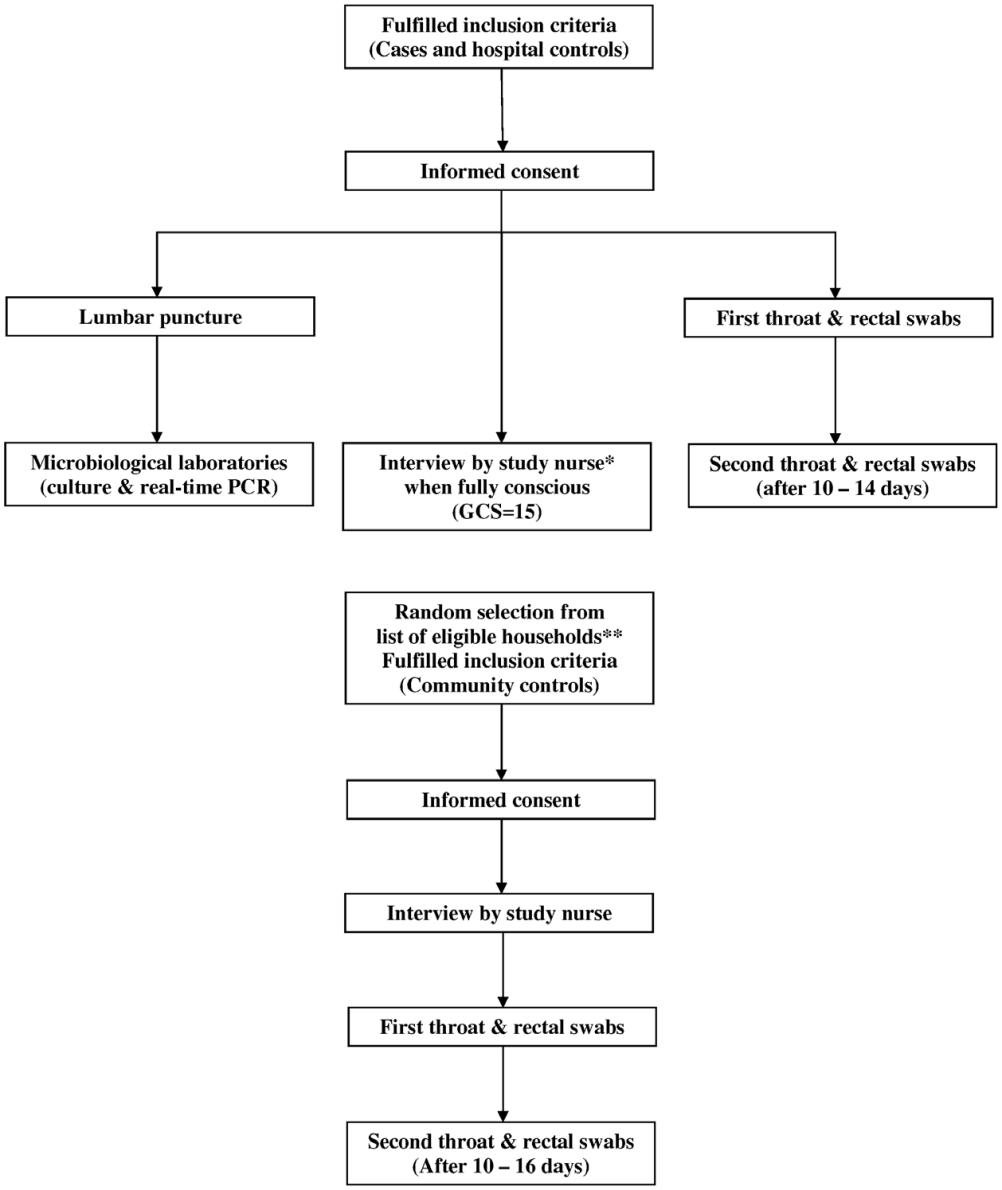
Flow diagram of recruitment of cases and controls. *Study nurses were unaware of case or control status of patients. ** Eligible households were defined as households in the same commune as a case.

Consecutive patients admitted with signs and symptoms consistent with central nervous system (CNS) infection were eligible for the study (Table 1). When *S. suis* infection was confirmed, patients were included as cases. After inclusion of a patient as a case, the next three consecutive patients admitted to the ward who met the inclusion criteria were included as hospital controls. Three community controls, matched for age (within a 10 year age range), were randomly identified from a list of eligible households available at the health center in the community of residence of the case, by using random number tables (Figure 1). Written informed consent was obtained from all patients and controls or their care takers.

**Table 1:** Inclusion and exclusion criteria.

Inclusion criteria	Exclusion criteria
**Cases:**	Did not provide informed consent.
At least 15 year-old	Recent history of bacterial meningitis (<1 year before admission).
*Streptococcus suis* meningitis or sepsis confirmed by blood culture, CSF culture, or CSF real-time PCR	Did not regain full consciousness (GCS 15) within 14 days after admission.
Admitted to CNS disease ward	Transferred to other hospitals within 7 days after admission
**Hospital controls:**	HIV positive.
At least 15 year-old	
Confirmed bacterial meningitis (not *S. suis*), eosinophilic meningitis, cryptococcal meningitis, viral encephalitis/meningitis* or malaria (confirmed by blood smear)	
Admitted to CNS disease ward	
**Community controls:**	
Living in the same commune as case for at least 4 weeks until inclusion of case	
Age matched with case (10 years range)	

(*) Viral encephalitis/meningitis was diagnosed on the basis of confirmation by positive diagnostic PCR or serology of CSF sample, or if the patient completely regained consciousness during treatment with antimicrobial agents for a duration of 48 hours or less.

### Assessment of risk factors

Risk factors were assessed using a standardized questionnaire. This questionnaire was developed in Vietnamese and validated at HTD and consisted of four parts; socio-demographic and cultural factors, medical history, potential exposure to pigs or pork and culinary habits and hygiene measures. We hypothesized that consumption of “high risk” food dishes (Table 2), potentially contaminated with *S. suis*, could function as a source of infection. The majority of the questions were “closed questions” but “open questions” which allowed participants to explain in their own words were also included. Patients and hospital controls were interviewed when they were fully conscious. The questionnaire was filled in by structured interview, which was carried out by one of two research nurses on the ward for cases and hospital controls, or at the residency of community controls. The interviewers were blinded towards the diagnosis of the patient in the case and hospital control groups.

**Table 2:** Definitions.

**Occupational exposures:** *at least one of the following occupations*
Butcher
Pig breeder
Slaughterer
Meat transporter
Meat processing
Veterinarian
Cook
**Contact with pigs/pork:** *at least one of the following contacts*
Bathe pigs
Feed pigs
Clean up the piggery
Slaughter pigs
Prepare or handle blood, organs from pigs
Visit a pig farm in the last 2 weeks
**“High risk” dishes:**
Pig/duck fresh blood
Pig tonsils/tongue
Pig stomach/intestines
Pigs uterus
Under-cooked pig blood
**Skin injuries:**
Patients were checked by nurses and doctors for skin injuries on forearms, hands and feet. Injuries were defined as lesions with signs of disruption of skin integrity.
**Underlying diseases:**
alcoholism, diabetes mellitus and splenectomy.
**Alcoholism:**
A person drinking beer >1500 ml/day or wine >250 ml/day in at least 5 days/week (Wine = SPIRIT 30–40°)
**Household exposure to pigs:**
breeding any number of pigs at home.
**Confirmed carriage:**
A person with 2 PCR positive swab samples on two separate occasions, at least 10–14 days in between
**Possible carriage:**
A person with 1 PCR positive swab sample

### Collection of swabs for detection of *S. suis* serotype 2 carriage

From all patients and controls, throat and rectal swab samples were taken on admission or at the community visit and a second set of swab samples was taken ten to fourteen days later. As detection of potential carriage in cases and hospital controls could be affected by their antimicrobial treatment, household members were also studied for carriage of *S. suis* serotype 2 since if carriage and associated transmission were to occur, household members of carriers are the most likely to become positive. Adult household members, defined as any adult (at least 15 years old) who resided for at least 50% of the week in the same house as the case or control, were identified either at the HTD, when taking care of a case or hospital control, or during the household visits. From each household, we choose a maximum of three adult household members from whom we took throat and rectal swabs in the same way as for the cases and controls, following written informed consent.

For all cases and controls who reported exposure to pigs at home, sampling of the pigs was performed in collaboration with the Sub-department of Animal Health of Ho Chi Minh City. Swab samples were taken from all pigs present (except pregnant sows to avoid stress-induced miscarriage) within 4 weeks of admission of the patient, or at the second visit to the community controls.

### Microbiological investigations

Culture of blood and cerebrospinal fluid was performed in the microbiology laboratory of the HTD using standard culture methods. Blood samples were taken on admission for all patients and blood cultures were performed using the BD BACTEC® 9050 blood culture system. *S. suis* was identified on the basis of colony morphology, negative catalase reaction, optochin resistance, and by APIStrep (Biomerieux, France) and subsequently serotyped (Statens Serum Institute, Denmark).

Swab samples were inoculated in transport medium (TRANSWABS®, UK) at the site, and transferred to the laboratory at HTD or stored at 4°C until transfer within 48 hours. Samples were inoculated into selective Todd-Hewitt broth (OXOID, UK), containing Streptococcal Selective Reagent (Oxoid) and crystal violet [8] and incubated overnight at 37°C, followed by real-time PCR for detection of *S. suis* serotype 2. Positive samples were cultured to retrieve *S. suis* isolates.

We used an internally controlled real-time PCR for detection of *S. pneumoniae*, *H. influenzae* type b, *N. meningitidis* and *S. suis* serotype 2 in CSF samples [4], [8], [9], [10]. The real-time PCR for detection of *S. suis* serotype 2 was also used on swab cultures. Previous validation of this PCR demonstrated a detection limit of 1–5 colony forming units per reaction [10].

After bacterial lysis, DNA was extracted using the EasyMag extraction system (BioMerieux, Ho Chi Minh City, Vietnam), according to manufacturer's instructions and subjected to consecutive monoplex PCR reactions (CSF samples).

### Sample size

Demographic data obtained during a randomized study on the efficacy of adjunct dexamethasone for the treatment of acute bacterial meningitis showed that 59/226 (26%) of the non-*S. suis* patients had potential occupational exposure to pigs compared to 46/78 (59%) of *S. suis* meningitis patients [3], corresponding to an odds ratio of 4. We decided on a target sample size of 100 cases (and 300 matched controls), corresponding to a recruitment period of approximately 4 years and a target odds ratio for 80% power of 2.1 assuming a probability of exposure in controls of 0.25 and a correlation coefficient for exposure between matched cases and controls of at most 0.2. With 100 cases, we also expected to fit reliable multivariate models with up to 10 covariates without over fitting the data [11].

### Statistical methods

All variables of interest were summarized by group (case, hospital, or community control). Categorical variables were summarized as number and percent (%). Continuous variables were summarized as median and interquartile range (IQR). To assess univariate associations of *S. suis* with potential risk factors in hospital controls, we used both logistic regression without any adjustment for covariates and with adjustment for sex, age, and living in a rural or urban area. We used conditional logistic regression for the matched community controls and these analyses were performed with and without additional adjustment for sex (in addition to the matched variables age and place of living). In a multivariate analysis of potential risk factors of main interest, all potential risk factors plus the potential confounders (e.g. age, sex, place of living) were jointly included in a logistic (hospital controls) or conditional logistic (community controls) regression model. No model selection such as backwards elimination was performed. We included separate effects of exposure to pigs or pork depending on whether the individual had skin injuries or not. *S. suis* infection occurred predominantly in males but controls were not matched by gender. As a sensitivity analysis, we therefore repeated the multivariate analysis including only male cases and controls. All analyses were performed with Stata version 10.1 (StataCorp) software.

## Results

**Figure 2:**
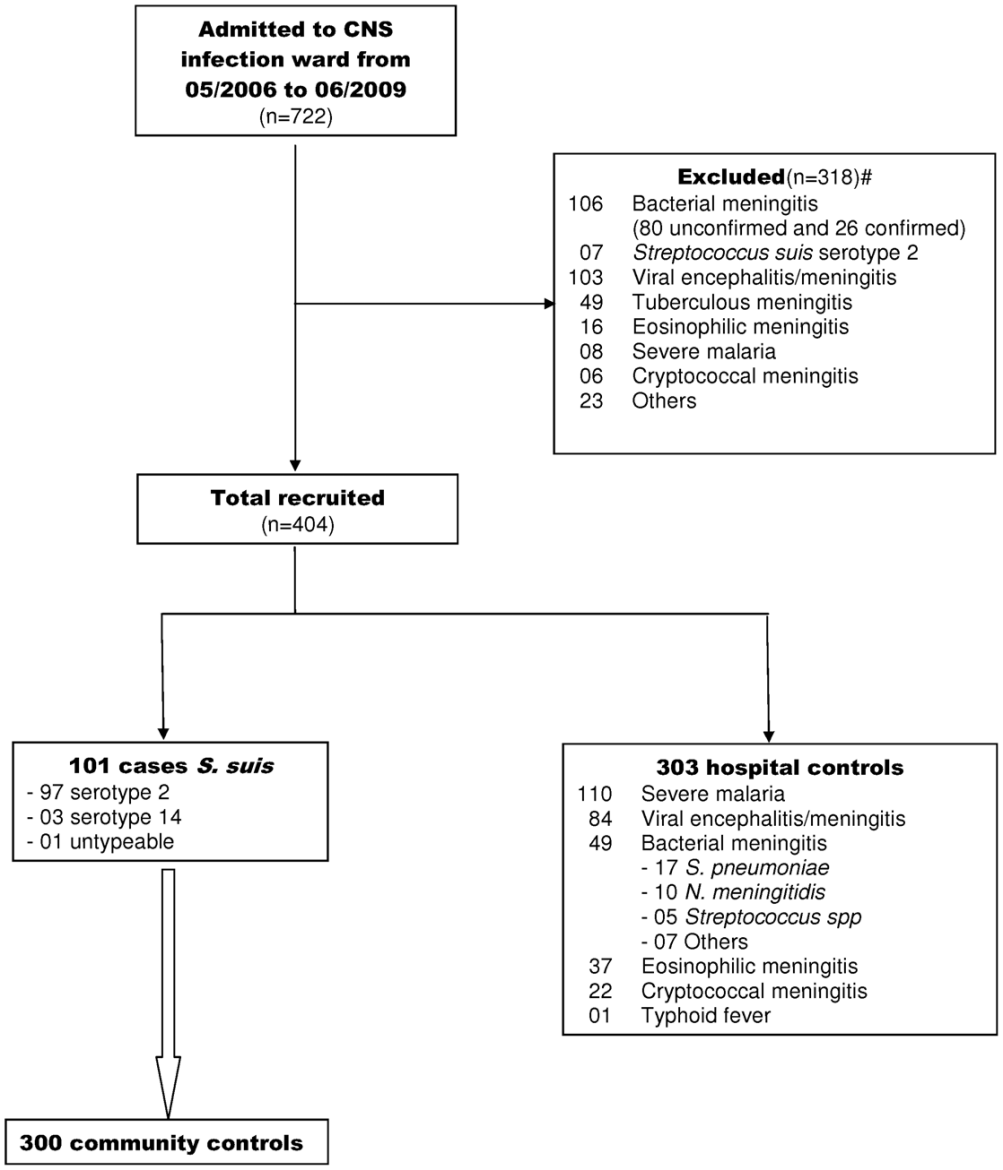
Flow diagram of inclusion of study participants. **^#^Reasons for exclusions of cases:** HIV (+) (3), transfer to other hospital because of presumed tuberculous meningitis (2), confusion more than 14 days after admission (1), language differences precluding interview (1). **^#^Reasons for exclusion of controls:** death (37), prolonged coma (72), unconfirmed bacterial meningitis (80), transfer to other hospitals (61), use of antimicrobial agents for more than 2 days in case of suspected viral encephalitis/meningitis (38), and absence of diagnosis of CNS infection (23). For one case, community controls could not be included because of too long distance of community to study site.

Between May 2006 and June 2009, 722 patients with suspected CNS infections or severe malaria were admitted to HTD. *S. suis* meningitis was diagnosed in 108 patients. Seven cases were excluded as they did not meet the inclusion criteria. We also excluded 311 other patients as they did not meet the inclusion criteria for hospital controls (Figure 2). It was not possible to recruit community controls at the residency of one *S. suis* case because of the distance from the study site (Central Viet Nam, 800 kms from Ho Chi Minh City). During the period of recruitment we therefore included 101 cases of confirmed *S. suis* meningitis, 303 hospital controls and 300 community controls for analysis (Figure 2).

### Characteristics of the participants

**Figure 3:**
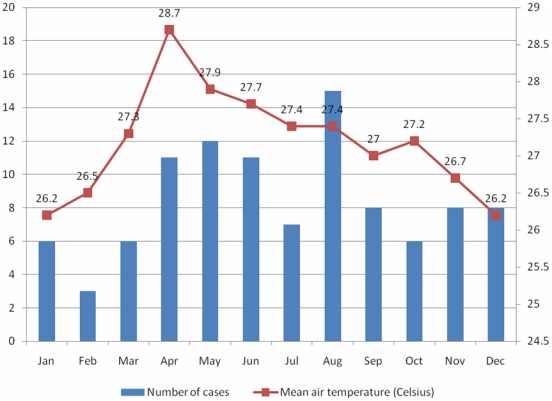
Distribution of *Streptococcus suis* meningitis cases during the study period. Distribution of *Streptococcus suis* meningitis cases and mean air temperature of southern Viet Nam in months during the study period (2006–2009) [17].

*S. suis* infection occurred sporadically throughout the year without any clear seasonality (Figure 3). Clustering of cases was not observed. Ninety-seven patients (96%) were infected with S. suis serotype 2 while only four patients (4%) were infected with other serotypes, including serotype 14 (3 cases) and untypeable serotype (1 case). *S. suis* cases were predominantly male (82%) and from a rural residence (81%) with a median (IQR) age of 50 (41–59) years. Twenty-one percent of cases had an occupation related to pigs, other exposure to pigs (46%), or reported eating “high risk” dishes in the two weeks prior to admission (48%). Hospital and community controls were more frequently female and (unmatched) hospital controls were significantly younger, with a higher proportion of urban residence (Table 3). We further analyzed the age distribution of cases, hospital controls and non-*S. suis* bacterial meningitis patients amongst the hospital controls. Nearly 70% of *S. suis* meningitis patients were older than 45 years, while only 25% of non-*S. suis* meningitis patients and 20% of all hospital controls belonged to this age group (Table 4). The 49 hospital controls with bacterial meningitis (not *S. suis*) were significantly younger than *S. suis* cases with median (IQR) age of 27 (23–45) compared to 50 (41–59) years (p<0.001).

**Table 3:** Characteristics of *Streptococcus suis* cases and controls.

Characteristics	Cases(n = 101)	Hospital controls(n = 303)	Community controls(n = 300)
**Sex, n(%)**			
Male	83 (82.2)	202 (66.7)	169 (56.3)
Female	18 (17.8)	101 (33.3)	131 (43.7)
**Residence, n(%)**			
Rural	82 (81.2)	193 (63.7)	243 (81)
Urban	19 (18.8)	110 (36.3)	57 (19)
**Age (years), median (interquartile range)**	50 (41,59)	27 (20,40)	50 (41,6)
**Occupations related to pigs** (1) **, n(%)**	21 (20.79)	8 (2.64)	8 (2.7)
**Education level, n(%)**			
Primary	52 (51.5)	96 (31.7)	175 (58.3)
Secondary “level 2”	30 (29.7)	134 (44.2)	73 (24.3)
Secondary “level 3”	11 (10.9)	40 (13.2)	30 (10)
University	1 (1)	21 (6.9)	2 (0.7)
Illiterate	6 (5.9)	12 (4)	19 (6.3)
**Religion**			
Buddhism	64 (63.4)	160 (52.8)	181 (60.3)
Catholicism	12 (11.8)	42 (13.9)	31 (10.3)
Christianity	1 (1)	4 (1.3)	0
Cao Dai	7 (7)	17 (5.6)	29 (9.7)
Other	2 (2)	1 (0.3)	6 (2)
No	15 (15)	77 (25.4)	53 (17.7)
**Ethnic backgroun**			
Kinh	99 (98.0)	287 (94.7)	298 (99.3)
Khmer	1 (1)	4 (1.3)	2 (0.7)
Chinese	1 (1)	4 (1.3)	0
Other	0	8 (2.6)	0
**Medical history, n(%)**			
Diabetes mellitus	3 (3)	3 (1)	4 (1.3)
Alcoholism	14 (13.9)	18 (5.9)	20 (6.7)
Splenectomy	1 (1	0	0
**Skin injuries, n(%)**	33 (32.7)	18 (5.9)	11 (3.7)
**Breeding pigs at home, n(%)**	23 (22.8)	33 (10.9)	41 (13.7)
**Any exposure to pigs/pork in the last 2 weeks, n(%)**	46 (45.5)	39 (12.9)	55 (18.3)
With skin injuries	20 (19.8)	5 (1.7)	2 (0.7)
Without skin injuries	26 (25.7)	34 (11.2)	53 (17.7)
**Eating any “high risk” dish in the last 2 weeks**	48 (47.5)	66 (21.8)	48 (16.)
Fresh pig blood	5 (5)	5 (1.7)	5 (1.7)
Tonsils/tongue	19 (18.8)	29 (9.6)	25 (8.3)
Stomach/intestines	45 (44.6)	53 (17.5)	42 (14)
Uterus	8 (7.9)	8 (2.6)	13 (4.3)
Undercooked pig blood	11 (10.9)	18 (5.9)	5 (1.7)
**Ill pigs at home in the last 4 weeks, x/n (%)**	10/23 (43.5)	1/33 (3.0)	0/40 (0)
**Pigs at home with ** ***S. suis*** ** serotype 2 (confirmed by PCR), x/n** (2) ** (%)**	9/22 (40.9)	3/13 (23.1)	5/28 (17.9)

(1) Butcher, pig breeder, slaughterer, roaster, meat transporter, meat processing, veterinarian and cook.

(2) Number of households with any number of PCR positive pig swab samples/ number of households where pigs where present and samples were taken.

**Table 4:** Age distribution of *Streptococcus suis* cases and hospital controls.

Age groups	Cases	Hospital controls	BM(1) (not *S. suis*) in hospital controls
**<30**	5 (5)	173 (57.1)	27 (55.1)
**30–44**	29 (28.7)	78 (25.7)	9 (18.4)
**45–59**	45 (44.6)	33 (10.9)	8 (16.3)
**60–74**	15 (14.9)	16 (5.3)	3 (6.1)
**75+**	7 (6.9)	3 (1)	2 (4.1)

(1) Bacterial meningitis.

### Analysis of risk factors

Occupations related to pigs, breeding pigs at home, exposures to pigs or pork with skin injuries, eating “high risk” dishes in the last 2 weeks and having ill pigs at home in the last 4 weeks were associated with *S. suis* meningitis, after adjustment for residency, age and sex (Table 5). *S. suis* infection was independently associated with occupations related to pigs, exposures to pigs or pork in the presence of skin injuries in the 2 weeks prior to infection, and eating “high risk” dishes in the 2 weeks prior to infection after multivariate analysis. These associations were found in comparisons of cases with the hospital control group as well as with the community control group. Breeding pigs at home, diabetes mellitus, alcoholism or exposure to pigs or pork without skin injuries were not associated with *S. suis* infection in multivariate analysis (Table 6). In a sensitivity analysis, which included only male cases and controls, exactly the same risk factors were significant with similar odds ratios as in the main analysis. Risk factors identified by multivariate analysis of male cases and controls from either control group included eating “high risk” dishes (OR_1_ = 3.46; 95%CI = [1.65–7.28] and OR_2_ = 4.79; 95%CI = [2.02–11.40]), occupations related to pigs (OR_1_ = 6.33; 95%CI = [1.55–25.79] and OR_2_ = 7.46; 95%CI = [1.56–35.74]), and exposures to pigs or pork in the presence of skin injuries (OR_1_ = 5.81; 95%CI = [1.07–31.48] and OR_2_ = 7.11; 95%CI = [1.00–50.54]).

**Table 5:** Risk factors of *Streptococcus suis* infection on univariate analysis.

Exposure	Cases versus Hospital controls				Cases versus Community controls			
	OR(1) (95%CI)	p value	OR(2) (95%CI)	p value	OR(1) (95%CI)	p value	OR(3) (95%CI)	p value
**Occupations related to pigs**	9.68(4.13–22.67)	<0.001	7.51(2.85–19.82)	<0.001	11.50(4.31–30.65)	<0.001	11.01(4.03–30.12)	<0.001
**Medical history**								
Diabetes mellitus	3.06(0.61–15.41)	0.175	0.82(0.13–5.23)	0.830	2.25(0.50–10.05)	0.288	3.75(0.75–18.73)	0.107
Alcoholism	2.55(1.22–5.33)	0.013	1.31(0.54–3.16)	0.547	2.50(1.15–5.45)	0.021	1.48(0.63–3.31)	0.381
**Skin injuries**	7.68(4.08–14.46)	<0.001	8.16(3.72–17.92)	<0.001	22.09(7.79–62.64)	<0.001	22.30(7.55–65.84)	<0.001
**Breeding pigs at home**	2.41(1.34–4.35)	0.003	2.34(1.09–5.00)	0.028	1.95(1.04–3.65)	0.036	1.99(1.04–3.80)	0.036
**Any exposure to pigs/pork in the last 2 weeks**	5.66(3.38–9.49)	<0.001	4.69(2.43–9.07)	<0.001	4.51(2.55–7.97)	<0.001	4.16(2.30–7.52)	<0.001
With skin injuries	14.72(5.36–40.42)	<0.001	12.16(3.74–39.50)	<0.001	30(7.01–128.35)	<0.001	26.95(6.14–118.23)	<0.001
Without skin injuries	2.74(1.55–4.86)	0.001	2.06(0.99–4.27)	0.052	1.66(0.92–3.00)	0.090	1.57(0.85–2.91)	0.152
**Eating any “high risk” dish in the last 2 weeks**	3.25(2.02–5.24)	<0.001	2.48(1.35–4.52)	0.003	6.00(3.33–10.81)	<0.001	4.38(2.72–8.08)	<0.001
**Ill pigs at home in the last 4 weeks** (4)	24.62(2.85–212.24)	0.004	30.10(2.72–333.64)	0.006	-	-	-	-
**Pigs at home with ** ***S. suis*** ** serotype 2 (confirmed by PCR)** (5)	2.31(0.49–10.82)	0.289	7.83(0.68–90.19)	0.099	-	-	-	-

(1) Crude OR based on logistic (hospital controls) or conditional logistic regression (community controls).

(2) Adjusted for age, sex and rural/urban residence, using logistic regression.

(3) Adjusted for sex (matched for age and residence), using conditional logistic regression.

(4) Only individuals with pigs at home were analyzed. OR could not be analyzed for community controls because none of them reported ill pigs at home.

(5) Only individuals who had pig swab samples at their houses were analyzed. OR could not be analyzed for community controls because there was no discordant pairs included in the analysis.

**Table 6:** Risk factors of *Streptococcus suis* infection - multivariate analysis.

Exposure	Cases versus Hospital controls		Cases versus Community controls	
	OR (95%CI)	p value	OR (95%CI)	p value
**Occupations related to pigs**	3.84 (1.32–11.11)	0.013	5.52 (1.49–20.39)	0.010
**Medical history**				
Diabetes mellitus	1.10 (0.17–7.31)	0.918	4.11 (0.78–21.68)	0.095
Alcoholism	1.02 (0.38–2.73)	0.969	0.72 (0.24–2.14)	0.553
**Breeding pigs at home**	1.02 (0.39–2.69)	0.965	0.83 (0.34–2.03)	0.681
**Any exposure to pigs/pork in the last 2 weeks**				
With skin injuries	7.48 (1.97–28.44)	0.003	15.96 (2.97–85.72)	0.001
Without skin injuries	2.15 (0.88–5.24)	0.092	1.14 (0.49–2.69)	0.757
**Eating any “high risk” dish in the last 2 weeks**	2.22 (1.15–4.28)	0.017	4.44 (2.15–9.15)	<0.001
**Rural**	2.39 (1.13–5.04)	0.022	-	-
**Age (by +10 years)**	2.59 (2.04–3.29)	<0.001	-	-
**Male sex**	4.47 (1.88–10.64)	0.001	3.53 (1.59–7.82)	0.002

“High risk” dishes predominantly consisted of undercooked food (Table 3). Exposure to pigs or pork in the presence of skin injuries, eating “high risk” dishes, or both, were reported in 72/101 *S. suis* cases (71.3%), 91/303 hospital controls (30%) and 84/300 community controls (28%). For 26 *S. suis* cases (25.7%), compared to 52 hospital controls (17.2%) and 29 community controls (9.7%), eating these “high risk” dishes was the only risk factor reported.

### Human carriage

To investigate potential carriage of *S. suis* serotype 2, 197 throat swab samples and 197 rectal swab samples were taken from 101 *S. suis* patients (Table 7). Six patients had PCR positive results, including one throat sample and six rectal samples. *S. suis* serotype 2 was cultured from this throat sample and from one of these rectal samples. Three of these six patients had pigs at home. No illness was reported in the last 4 weeks in these pigs, and the PCR results of pig tonsil swab samples were negative. None of these patients had skin injuries. Three patients had eaten pig intestines prior to admission. Of these, the throat swab sample was positive in one patient and rectal swab samples were positive in the two others. One patient, who had eaten pig intestines two days before admission, had two PCR positive rectal swab samples on separate occasions. *S. suis* serotype 2 was cultured from the first sample.

**Table 7:** Results of *Streptococcus suis* serotype 2 PCR of throat and rectal swabs.(1)

Samples	Cases		HM(2)/cases		Hospital controls		HM/hospital controls		Community controls		HM/community controls	
	1^st^	2^nd^	1^st^	2^nd^	1^st^	2^nd^	1^st^	2^nd^	1^st^	2^nd^	1^st^	2^nd^
Throat	1/101	0/96	0/205	0/182	0/302	0/279	0/291	0/212	0/300	0/291	0/424	0/347
Rectum	5/101	1/96	0/204	0/181	0/302	0/279	0/291	0/211	0/300	0/291	0/422	0/340

(1) Number positive/total number tested (each person had 2 samples taken on 2 separate occasions with a minimum of 10–14 days in between).

(2) Household members.

We collected 1162 throat and rectal swab samples from the 303 hospital controls and 4492 throat and rectal swab samples from healthy persons, including 300 community controls and 920 household members of cases, hospital controls and community controls. In none of these samples was S. *suis* serotype 2 detected. (Table 7).

### Pig carriage

We collected 571 pig swab samples from pigs present around the house of 22 of 23 cases, 28 of 41 community controls and 13 of 33 hospital controls respectively, who kept pigs around the house (Table 3). The median herd size was 7 pigs (range, 1 to 50). *S. suis* serotype 2 was detected in 9 (41%) case group herds, 3 (23%) hospital control group herds, and 5 (18%) matched community control group herds. Differences between case group and control groups were not statistically significant (Table 5).

## Discussion

We conducted the largest prospective epidemiological assessment of risk factors of *S. suis* infection globally. In addition to the previously suggested risk factors, occupational exposure and contact with pigs or pork without skin protection, we identified the ingestion of food with a high risk of contamination with *S. suis* serotype 2 to be an important risk factor for *S. suis* meningitis. To investigate eating habits as a risk factor of *S. suis* infection, we focused on potential “high risk” dishes common in Vietnam. These include fresh or under-cooked blood, tonsils, tongue, stomach, intestines and uterus. Such food items typically are undercooked when eaten as a main dish (as opposed to as components of well cooked main dishes such as rice or noodle soups), as was generally the case in our patients. By multivariate analysis, we demonstrated that eating these “high risk” dishes in the 2 weeks prior to admission was a significant risk factor for this infection. Eating habits were also confirmed as a risk factor during a relatively small *S. suis* outbreak in Thailand, associated with consumption of fresh pig blood. [12]. Eating pork was not associated with *S. suis* cases in a matched case-control study conducted during the outbreak in Sichuan province in 2005 [13]. *S. suis* lives as normal flora in the respiratory, gastrointestinal and genital tract of pigs and can cause invasive disease in pigs. *S. suis* was isolated from raw pork samples obtained from markets in Hong Kong [14]. There may be a high bacterial load in food items that are kept at high ambient temperatures. Therefore, patients may be infected with *S. suis* through gastrointestinal tract if the “high risk” dishes are served as raw or under-cooked food.

We observed that the mean age of *S. suis* meningitis patients was significantly higher than the age of patients with bacterial meningitis caused by other bacteria, and that high age was associated with increased risk of infection with *S. suis*. In contrast, pediatric infections with *S. suis* are extremely rare, presumably related to a lack of exposure associated with increased risk of *S. suis* infection, in children.

The association between human *S. suis* infection and occupational exposures to pigs or pork has been reported in Europe and Asia since 1968 [6], [15], [16]. In Vietnam, the proportion of patients reported to have occupational exposures was lower than reported in European patients but it remained an important independent risk factor. Slaughtering and processing sick or dead pigs were also associated with *S. suis* infection in a case-control study conducted during the Sichuan outbreak [13].

Significant skin injury was evident in 5/35 (14%) of people with *S. suis* in the UK, 4/15 cases (16%) in Hong Kong and 104/215 cases (48%) in Sichuan province's outbreak [2], [7], [15]. In our study, skin injuries were reported in 33/101 (33%) of *S. suis* patients compared to 18/303 (6%) of hospital controls and 11/300 (4%) of community controls (Table 3). These minor skin injuries may allow direct entry of the bacteria in people with direct contact with infected pigs or pork. Contact with pigs within the last two weeks in the presence of skin lesions was associated with a significant high risk of infection (Table 6). Skin injuries were most often recorded in slaughter house workers, cooks and housewives involved in processing meat. Skin protection, including gloves, hand washing and exclusion of people with obvious skin lesions from direct contact with pigs and pork meat, may help to reduce the incidence of the disease.

We were unable to demonstrate *S. suis* serotype 2 carriage in 1522 healthy persons or patients without *S. suis* infection, including those with pig exposures. In contrast, 6/101 (6%) of patients had PCR positive swab samples, a rate similar to what was found in slaughterhouse workers in Germany [16]. Eating pig intestines in the few days prior to admission was reported in 3/6 of the patients with a PCR positive throat or rectal swab, two of which were also culture positive. Taken together, rather than indicating human carriage of *S. suis* serotype 2, our results strengthen the hypothesis that the gastrointestinal tract may be a route of entry for at least a proportion of patients.

In conclusion, *S. suis* is an important and emerging public health issue in Asia and one with the potential for both endemic transmission and for explosive epidemics. We identified risk factors for *S. suis* infection which can be addressed in health education programs targeted at individuals and communities at risk, focusing on skin protection for those in direct contact with pigs or pork and avoiding eating raw or under-cooked pig products.
