# Correction: Risk Factors of *Streptococcus suis* Infection in Vietnam. A Case-Control Study

**DOI:** 10.1371/annotation/25743e50-5a58-4fb6-b466-9a345311d4a8

**Published:** 2012-05-15

**Authors:** Dang Trung Nghia Ho, Thi Phuong Tu Le, Marcel Wolbers, Quang Thai Cao, Van Minh Hoang Nguyen, Vu Thieu Nga Tran, Thi Phuong Thao Le, Hoan Phu Nguyen, Thi Hong Chau Tran, Xuan Sinh Dinh, Song Diep To, Thi Thanh Hang Hoang, Truong Hoang, James Campbell, Van Vinh Chau Nguyen, Tran Chinh Nguyen, Van Dung Nguyen, Thi Hoa Ngo, Brian G. Spratt, Tinh Hien Tran, Jeremy Farrar, Constance Schultsz

The byline published in the correction is incorrect. The correct byline is: Ho Dang Trung Nghia, Le Thi Phuong Tu, Marcel Wolbers, Cao Quang Thai, Nguyen Van Minh Hoang, Tran Vu Thieu Nga, Le Thi Phuong Thao, Nguyen Hoan Phu, Tran Thi Hong Chau, Dinh Xuan Sinh, To Song Diep, Hoang Thi Thanh Hang, Hoang Truong, James Campbell, Nguyen Van Vinh Chau, Nguyen Tran Chinh, Nguyen Van Dung, Ngo Thi Hoa, Brian G. Spratt, Tran Tinh Hien, Jeremy Farrar, Constance Schultsz. 

